# Temperature-dependent egg production and egg hatching rates of small egg-carrying and broadcast-spawning copepods *Oithona similis*, *Microsetella norvegica* and *Microcalanus pusillus*

**DOI:** 10.1093/plankt/fbaa039

**Published:** 2020-09-07

**Authors:** Coralie Barth-Jensen, Marja Koski, Øystein Varpe, Peter Glad, Owen S Wangensteen, Kim Præbel, Camilla Svensen

**Affiliations:** FACULTY OF BIOSCIENCES, FISHERIES AND ECONOMICS, UIT THE ARCTIC UNIVERSITY OF NORWAY, TROMSø, NORWAY; NATIONAL INSTITUTE FOR AQUATIC RESOURCES, TECHNICAL UNIVERSITY OF DENMARK, LYNGBY, DENMARK; NORWEGIAN INSTITUTE FOR NATURE RESEARCH, BERGEN, NORWAY; DEPARTMENT OF BIOLOGICAL SCIENCES, UNIVERSITY OF BERGEN, BERGEN, NORWAY; FACULTY OF BIOSCIENCES, FISHERIES AND ECONOMICS, UIT THE ARCTIC UNIVERSITY OF NORWAY, TROMSø, NORWAY; FACULTY OF BIOSCIENCES, FISHERIES AND ECONOMICS, UIT THE ARCTIC UNIVERSITY OF NORWAY, TROMSø, NORWAY; FACULTY OF BIOSCIENCES, FISHERIES AND ECONOMICS, UIT THE ARCTIC UNIVERSITY OF NORWAY, TROMSø, NORWAY; FACULTY OF BIOSCIENCES, FISHERIES AND ECONOMICS, UIT THE ARCTIC UNIVERSITY OF NORWAY, TROMSø, NORWAY

**Keywords:** female carbon content, hatching success, low temperature, seasonality, weight-specific egg production rate

## Abstract

Reproductive rates of copepods are temperature-dependent, but poorly known for small copepods at low temperatures, hindering the predictions of population dynamics and secondary production in high-latitude ecosystems. We investigated egg hatching rates, hatching success and egg production of the small copepods *Oithona similis* and *Microsetella norvegica* (sac spawners) and *Microcalanus pusillus* (broadcast spawner) between March and August. Incubations were performed at ecologically relevant temperatures between 1.3 and 13.2°C, and egg production rates were calculated. All egg hatching rates were positively correlated to temperature, although with large species-specific differences. At the lowest temperatures, *M. pusillus* eggs hatched within 4 days, whereas the eggs from sac spawners took 3–8 weeks to hatch. The egg hatching success was ≤25% for *M. pusillus*, >75% for *O. similis* and variable for *M. norvegica*. The maximum weight-specific egg production rate (μg C μg^−1^ C d^−1^) of *M. pusillus* was higher (0.22) than *O. similis* (0.12) and *M. norvegica* (0.06). *M. norvegica* reproduction peaked at 6–8°C, the prevailing *in situ* temperatures during its reproductive period. The difference in reproductive rates indicates species-specific thermal plasticity for the three copepods, which could have implications for present and future population dynamics of the species in arctic fjords.

## INTRODUCTION

Small copepods, such as the cosmopolitan *Oithona* spp., are numerically abundant (Ormańczyk *et al.,* 2017; Poulsen and Kiorboe, 2006; Schnack-Schiel, 2001; Zamora-Terol *et al.,* 2013), and can seasonally dominate copepod biomass at high latitudes (Arendt *et al.,* 2013; Svensen *et al.,* 2011). They are an important food source for early larval stages of fish and carnivorous zooplankton, and can serve as an alternative or complementary carbon source for older larval stages (Castellani *et al.,* 2007; Grønkjær *et al.,* 2018; Turner, 2004). Some small copepod species are important for biogeochemical cycles (Koski *et al.,* 2017; Turner, 2004), including the biological carbon pump, both through their diet (e.g. feeding on suspended particles and microzooplankton) and their sloppy feeding behavior (Shoemaker *et al.,* 2019; Svensen and Vernet, 2016). Most small copepod species are active year round (Madsen *et al.,* 2008; Zamora-Terol *et al.,* 2014) while the large copepod species such as *Calanus* spp. hibernate in winter (Conover, 1988).

A body size of <2 mm defines small copepod species (Roura *et al.,* 2018), but these species are not functionally uniform (Litchman *et al.,* 2013). There is large variability in their feeding behavior (Drits and Semenova, 1984; Nishibe *et al.,* 2010), reproductive strategies (Kiørboe and Sabatini, 1994) and seasonal population dynamics (Arendt *et al.,* 2013; Ashjian *et al.,* 2003; Madsen *et al.,* 2008). At temperate and high latitudes, small copepods have been suggested to increase in abundance relative to larger ones due to climate-induced changes in salinity (Mäkinen *et al.,* 2017) and temperature (Beaugrand *et al.,* 2002; Coyle *et al.,* 2008; Eisner *et al.,* 2014). For example, small copepods tend to have increased production in warmer and more stable surface waters (Coyle *et al.,* 2008; Mäkinen *et al.,* 2017).

Measurements of growth (Uye *et al.,* 2002) or egg production (Zamora-Terol *et al.,* 2014) are commonly used to understand population dynamics and to estimate secondary production of copepods. These measurements are species-specific and time-consuming to obtain (Avila *et al.,* 2012), and limited knowledge is available on small copepods growth and reproductive rates (Madsen *et al.,* 2008; Norrbin, 1991; Turner, 2004). For the understudied species, rates from similar-sized or taxonomically close species are often used (e.g. Madsen *et al.,* 2008; Middelbo *et al.,* 2019; Nielsen and Andersen, 2002). However, the same environmental forcing may have different effects on different species, even when they belong to the same genus (Eisner *et al.,* 2014; Ershova *et al.,* 2016; Ershova *et al.,* 2017; Jónasdóttir, 1989). Estimation of copepod secondary production based on average rates of model species rather than the dominant (but perhaps understudied) ones may therefore result in erroneous estimates.

A species response to increasing temperature is an important facet of environmental ecophysiology, with phenotypic plasticity being the capacity of organisms to modify their morphology, physiology or life history under environmental influence (Booth *et al.,* 2018; Calow, 2009; Ortega-Mayagoitia *et al.,* 2018). Thermal plasticity is attributed to temperature-induced modifications and can result in local adaptation in copepods (Drillet *et al.,* 2008; Lonsdale and Levinton, 1986). Water temperature in the Arctic is predicted to rise over the next decades (Alexander *et al.,* 2018). To assess the present state of the community and a future possible shift from large to small copepod species, more knowledge is needed about the temperature dependence of the vital rates of small copepod species. Temperature-dependent reproductive rates of copepods include the egg hatching rate (Ambler, 1985; Andersen and Nielsen, 1997) and egg production (Bunker and Hirst, 2004; Huntley and Lopez, 1992) whereas the clutch size and hatching success have been described as both temperature-dependent (Devreker *et al.,* 2012; Ershova *et al.,* 2016; Hansen *et al.,* 2010) and temperature-independent (Dvoretsky and Dvoretsky, 2009a; Ershova *et al.,* 2016; Kurbjeweit, 1993; Weydmann *et al.,* 2015). The latency time, i.e. the time between the separation of hatched eggs from the female to the production of a new egg sac (Devreker *et al.,* 2012), seems to be temperature-independent for some small copepod species (Uye *et al.,* 1982; Uye *et al.,* 2002; Uye and Sano, 1995; Ward and Hirst, 2007), but was described as temperature-dependent for other species (Devreker *et al.,* 2012). However, few studies have measured the reproductive rates of small copepods at low temperatures, although a broad range of life history adaptions could be expected in response to the highly seasonal environment of high-latitude seas (Varpe, 2017).

In the present study we investigated three small copepod species: *Oithona similis* (Cyclopoida), *Microsetella norvegica* (Harpacticoida) and *Microcalanus pusillus* (Calanoida)*.* All three species are abundant in sub-arctic Balsfjord (69°N; northern Norway), have comparable body size (~500 μm), but differ regarding life-history strategies (Benedetti *et al.,* 2016; Brun *et al.,* 2017). Copepods have two main reproductive strategies; broadcast spawners (or free spawners) release a relatively large number of eggs (Kiørboe and Sabatini, 1994), whereas egg-carrying copepods (or sac spawners) produce fewer eggs clustered in one or two egg pouches. Although Kiørboe and Sabatini (1994) compared the reproductive strategies of sac spawners and broadcast spawners, their dataset include few small copepods, mainly from the genus *Oithona* that is the most investigated small copepod (e.g. Mironova and Pasternak, 2017; Nielsen *et al.,* 2002; Sabatini and Kiørboe, 1994; Zamora-Terol *et al.,* 2014). In contrast, the reproduction and population dynamics of the egg-carrying *M. norvegica* (Koski *et al.,* 2014; Mironova and Pasternak, 2017; Svensen *et al.,* 2018; Uye *et al.,* 2002) and broadcast spawner *M. pusillus* have been scarcely investigated (Norrbin, 1991).

We investigated the temperature-dependent responses of reproductive rates in *O. similis*, *M. norvegica* and *M. pusillus*, expecting that egg hatching rates of the three species will increase with temperature within their tolerance range. We also compared the influence of temperature on the egg hatching success and egg production of these ubiquitous species with different reproductive strategies. Genetic tools have revealed that cryptic or pseudocryptic species may be relatively common in marine species, including copepods (Lajus *et al.,* 2015). It is therefore unsure if the historically reported broad tolerance ranges within a certain morphologically identified species can still be trusted for single species (Knowlton, 1993). The *Microcalanus* species identification was therefore resolved through genetic analysis. For *O. similis*, several lineages have been described, but only one was found in the Arctic (Cornils *et al.,* 2017). We can therefore assume that the *O. similis* specimens in the present study belonged to the same species lineage.

## MATERIAL AND METHODS

We investigated egg hatching rates, egg development times and hatching success of *O. similis*, *M. norvegica* and *M. pusillus* as a function of temperature, within the range of 1.3–13.2°C. In total, 22 incubations were conducted (Table I). The temporal spread of the incubations covered a wide temperature range so that the copepods response to different temperatures could be studied without needing a prior acclimation period.

**Table I:** Overview of the incubations including the start date, in situ minimum and maximum temperatures at the depths from 170 to 0 m, incubation temperature (mean ± SD) and number of females used in each incubation

Start date	Temperature (*in situ*, °C)	Temperature (incubation, °C)	*Oithona similis*	*Microsetella norvegica*	*Microcalanus pusillus*
9.06.2017	4.8–11.4	4.6 ± 0.1	60 (H^*1^)	60 (H^*1^)	
19.06.2017	5.0–9.8	7.9 ± 0.2	61 (H^1^)	57 (H^*^)	
		11.3 ± 0.1	65 (H)		
15.08.2017	Surface 9.0	10.4 ± 0.1		30 (H)	
		13.2 ± 0.2	30 (H)	30 (H)	
1.03.2018	1.7–2.1	1.3 ± 0.1	10 (H^2^)		
12.03.2018	1.2–1.8	1.3 ± 0.1	30 (H^3^)		
3.05.2018	2.0–3.5	4.0 ± 0.1		30 (H)	30 (H, EP)
		7.0 ± 0.2		30 (H)	30 (H, EP)
11.06.2018	2.5–7.7	3.0 ± 0.1		30 (H)	30 (H, EP)
		4.8 ± 0.1		30 (H)	29 (H, EP, DNA)
		5.7 ± 0.1		30 (H)	30 (H, EP, DNA)
24.08.2018	6.8–10.2	6.1 ± 0.2		30 (H)	
		9.8 ± 0.1			30 (H, EP)

H, hatching; EP, egg production; DNA, DNA sequencing of the female *Microcalanus* used in the incubation. ^*^both attached and detached egg sacs, (blank) no experiment. Duration was 7 days for all experiments except for: ^1^11 days, ^2^15 days and ^3^18 days.

### Sampling

Copepods were collected in June and August 2017, and in March, May, June and August 2018 (Table I) at Svartnes, Balsfjord, Norway (N: 69° 22.947′; E: 19° 05.414′, depth 180 m). Balsfjord is one of the coldest fjords in Norway (Hopkins *et al.,* 1989), with mean surface temperature ranging from 1.3°C in February to 8.6°C in July and August (Eilertsen and Skarðhamar, 2006). A WP-2 net (64 or 90 μm-mesh, Hydro-Bios, Germany, 0.25 m^2^ opening), equipped with a non-filtering cod end, was raised at 0.3–0.4 m s^−1^ from 50 m (2017) or 100 m (2018) to the surface. On deck, the content of the cod end was placed in 20 L of surface seawater, and transported to the laboratory within 2 h. Copepod samples were stored at 8°C for ~8 h during the experimental set-up, and the handling time was minimized for incubations with temperatures that deviated most from 8°C. *In-situ* temperature of the water column was obtained using conductivity, temperature, depth (CTD) profiler (Seabird model 25 Sealogger). Water samples were collected at four depths (5, 20, 50 and 150 m) using 20 L Go-Flo bottles (General Oceanics, Florida, USA). Water samples were stored in acid-washed Nalgene bottles in a dark cooler for 3 h until arrival at the laboratory. For each depth, three 100 mL aliquots were filtered for total chlorophyll *a* (Chl *a*, GF/F filter, 0.7 μm). The filters were extracted in 5 mL methanol for 12–18 h at 4°C in the dark (modified from Strickland and Parsons, 1972). Chl *a* was measured with a fluorometer (10-AU, Turner Designs, California, USA), and concentrations for the three aliquots were averaged for each depth.

### *Oithona similis* and *Microsetella norvegica* egg-hatching incubations

Egg hatching was investigated at temperatures between 1.3°C and 13.2°C for *O. similis*, and at temperatures between 3.0°C and 13.2°C for *M. norvegica*. With the exception of August 2017 and May 2018, incubation temperatures reflected the *in situ* temperature at the time of sampling (Table I). Incubation temperatures were 3.5 and 4.2°C above *in situ* temperatures in May 2018 and August 2017, respectively. These higher temperatures were necessary to cover a 10°C temperature range. All incubations were performed without acclimation of the animals, following the procedure of Nielsen *et al.* (2002).

Uye *et al.* (2002) removed egg sacs manually from females *M. norvegica* and incubated them separately. This procedure is not usual for other egg-carrying copepods, including *O. similis,* where females and eggs are typically incubated together. In our first incubations, we therefore tested whether hatching rates of attached vs. detached egg sacs differed. For both species, we sorted 60 egg-carrying females using a stereomicroscope. The egg sacs were detached from 40 females, while 20 females were incubated with their egg sacs attached. Single females with their egg sacs or single egg sacs were individually incubated for 12 days, in order to ensure that all eggs had sufficient time to hatch. Since there were no significant differences in hatching rates between the two methods (Mann–Whitney rank-sum test, *P* ≥ 0.424), the egg sacs were not separated from the females in the remaining incubations.

The duration of each incubation at the different temperatures was determined on the basis of the first incubations at 4.6°C, which lasted for 12 days. In these incubations, all viable eggs hatched within 11 days (*O. similis*) or within 4 days (*M. norvegica*). A change of color of the eggs was interpreted as a sign of degradation (Burkart and Kleppel, 1998; Drillet *et al.,* 2011), and discolored eggs were assumed to be unviable. For incubations at higher temperature, we assumed that 7 days would be sufficient as this represented the median duration of egg hatching of both species at 4.6°C. The two incubations at 1.3°C (*O. similis*, Table I) were prolonged to 15 and 18 days, in accordance with published data (Nielsen *et al.,* 2002). For all incubations, females with egg sacs were placed individually into 2.5 mL of 0.2 μm filtered seawater (FSW; Halvorsen, 2015), in 12-well culture plates and incubated in temperature-controlled incubators (Termaks KB8182, Termaks, Norway). The experimental design by Nielsen *et al.* (2002) relies on an even spread of the females through their egg-carrying cycle. We aimed for a minimum of 30 females per incubation, unless the total abundance of females in the sample was <30 (Table I). The water temperature was logged (model Kistock, Kimo, France) every 5 min for the duration of the incubation. We defined the number of eggs carried in the egg sacs of a female as a clutch. Clutch size was obtained for each female at the beginning of incubations by counting the number of eggs carried. Every 24 h (for incubations at 1.3°C) or 8 h (for all other temperatures), the wells were checked for hatching following gentle mixing of the water. Every second day ~50% of the water was replaced with fresh FSW. A hatching event for the entire clutch was defined as the time when at least one freely swimming nauplius was observed in the well. After the hatching event was recorded, the well was monitored to determine the final number of hatching eggs. Newly hatched nauplii were removed. The handling time was <10 min/plate. Wells containing a dead female during the first 24 h of the incubation were excluded from the dataset. During the first 2 days of the incubation, all females were photographed using a camera (Leica DFC450) connected to a stereomicroscope (Leica MZ16, ×84–100 magnification) for measurements of prosome length (for *O. similis*) or total length (for *M. norvegica*). In addition, 50 eggs of each species (×100–110 magnification) were photographed and measured with a precision of ±7 μm.

### *Microcalanus pusillus* egg production and hatching rate

Incubations of *M. pusillus* were conducted in May, June and August 2018. For each incubation, 29 to 30 females were randomly selected from the samples (Table I) and individually incubated for 24 h in 2.5 mL of 0.2 μm-FSW in 12-well culture plates, under similar conditions to those used for *O. similis* and *M. norvegica*. Overall mortality within the 24-h incubations never exceeds 7%, except for 23% mortality in the 9.8°C incubation. After 24 h, the eggs in each well (the clutch) were counted. The average clutch size for *M. pusillus* excluded the non-producing females. Females were photographed and size measured, before being preserved in pure grade ethanol (96%). The clutches were returned to the incubators and followed for 6 days. Eggs were checked for hatching every 8 h, with a handling time of maximum 10 min per plate. The same definition of a hatching event was used for *M. pusillus* as for the sac spawners. The mean development time (D, d) refers to the time between egg production and egg hatching and was calculated as the mean of all hatching events in all wells incubated at the same temperature.

### Species determination of *M. pusillus*

It is uncertain whether one or two *Microcalanus* species are present in Balsfjord: *M. pygmaeus* and/or *M. pusillus* (S. Kwaśniewski, personal communication). The species can be identified on the basis of differences in the terminal spines on the second and fourth exopods, and from differences in the antennule/prosome length ratio (Koszteyn *et al.,* 1991). Use of both methods is challenging on live specimens and species identification could not be conducted prior to the egg incubations. We employed the length ratio method for all specimens after fixation in ethanol, using a stereomicroscope at ×100 magnification. Additionally, 58 females from two incubations (4.8°C and 5.7°C in June 2018) were sequenced to confirm taxonomic identification. DNA was extracted from individual females following a modified version of the HotShot protocol (Meissner *et al.,* 2013; Truett *et al.,* 2000). The Leray fragment of the mitochondrial cytochrome c oxidase subunit I (COI) was amplified using tagged mlCOIintF-XT 5′-GGWACWRGWTGRACWITITAYCCYCC-3′ as forward primer and tagged jgHCO2198 5′-TAIACYTCIGGRTGICCRAARAAYCA-3′ as reverse primer, and the polymerase chain reaction (PCR) was performed with conditions described in Wangensteen *et al.* (2018). Multiplexed libraries for next generation sequencing were obtained using the NEXTflex PCR-free DNA-seq kit (BIOO Scientific, TX, USA) and sequencing was performed on an Illumina MiSeq using a nano-kit V2 2x250 bp (Illumina, CA, USA) following the manufacturer’s protocol. The obtained paired-end reads were aligned, demultiplexed, quality-filtered, and dereplicated using a custom pipeline based on OBITools (Boyer *et al.,* 2016), following procedures described in Ershova *et al.* (2019). The most abundant sequence obtained from each individual was compared to available sequences in BOLD (barcode reference database) for *M. pusillus* and sequences of *M. pygmaeus* (T. Falkenhaug, Institute of Marine Research, Norway). The morphological identification of the 58 females was then compared to their genetic identification to check for the robustness of the species identification.

### Copepod carbon content

The particulate organic carbon (POC) contents of female *O. similis, M. norvegica* and *M. pusillus* were analyzed for samples collected in August 2016, February, March and April 2017 and June 2018. Between 60 and 300 females (without egg sacs) were sorted under a stereomicroscope (Leica MZ16, ×64–80 magnification), rinsed in 0.2 μm-FSW and placed onto precombusted GF/F filters (450°C, 0.7 μm pore size). The filters were stored frozen at −20°C until analysis. Prior to analysis, the filters were dried (60°C) and thereafter fumed with concentrated HCl (12 mol L^−1^) to remove inorganic carbon. The filters with the females were analyzed using a CHN Lab Leeman 440 elemental analyzer. Measured values of POC for blanks (filters without copepods) were subtracted from filters containing copepods. Due to the loss of the June carbon measurement, *M. norvegica* carbon weight was approximated as the average between the April and August measurement for this month.

## CALCULATIONS AND STATISTICAL ANALYSIS

Five variables were extracted from egg incubations with *M. norvegica* and *O. similis*: hatching rate of eggs, hatching success of clutches, hatching success of eggs in each clutch, total egg hatching success and weight-specific daily egg production.

### Egg hatching rate

To obtain the egg hatching rate, the cumulative hatching events of the sac spawners were plotted against the incubation time. The large number of females incubated was assumed to ensure an even spread of the females’ egg-carrying cycle (Nielsen *et al.,* 2002), which resulted in a linear increase of hatching events with time. The egg hatching rate (HR, d^−1^) was defined as the slope of this linear regression between the cumulative hatching events and the incubation time. The regressions were forced through the origin as no females with already hatched clutches were incubated at T_0_. Hatching events for the broadcast spawner *M. pusillus* were rather synchronous in a single incubation, as clutches were all produced within 24 h. Therefore, the estimation of the egg hatching rate was not determined by linear regression, but as the reciprocal of the mean development time (D, d), for all hatching events within a single incubation.

### Hatching success

The hatching success of clutches (HS_C_, %) for each incubation was estimated as a percentage of clutches with at least one hatching event. The hatching success of eggs in each clutch (HS_E_, %) was derived from the same incubation. This was expressed as the percentage of eggs in each clutch that had hatched by the end of the incubation. Total egg hatching success (HS_T_, %) was then calculated by multiplying HS_C_ by HS_E_. These variables were calculated in the same way for the three species.

### Egg production

For *M. pusillus*, egg production (eggs female^−1^ d^−1^) was estimated as the total number of eggs produced in 24 h divided by the number of females (including the non-producing females). Population-specific egg production could not be estimated for *O. similis* and *M. norvegica*, as the *in situ* ratio of females with egg sacs to the females without eggs was unknown. However, we estimated the individual carbon-specific egg production of the ovigerous (or reproducing) females (SEP_OV_, μg C μg^−1^ C d^−1^) for all three species, assuming that the latency time would be short and not temperature-dependent (Uye *et al.,* 2002; Uye and Sano, 1995). The SEP_OV_ was thus calculated by multiplying the average clutch size by the temperature-specific egg hatching rate obtained from the hatching incubations and the egg to female carbon ratio as:


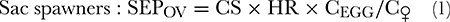



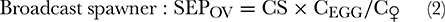


where CS is the average clutch size (# eggs female^−1^), HR is the estimated hatching rates (d^−1^), C_EGG_ is the carbon content of an egg (μg C), and C_♀_ is the carbon content of a female (μg C).

Egg hatching rate for the sac spawners was calculated using the surface temperature (depending on the sampling date, Table I). The carbon content of females was measured at different times of the year, and the value closest in time to the incubation was used. Egg carbon content was calculated based on volumes (calculated from diameters), converted to carbon using the conversion 0.14 × 10^−6^ μg C μm^−3^ for *O. similis* and *M. pusillus* (Kiørboe *et al.,* 1985; Sabatini and Kiørboe, 1994). *M. norvegica* eggs are spherical or ovoid (Uye *et al.,* 2002), and their egg volume was calculated from length and width measurements and converted to carbon using 0.19 × 10^−6^ μg C μm^−3^ (Uye *et al.,* 2002).

**Fig. 1:**
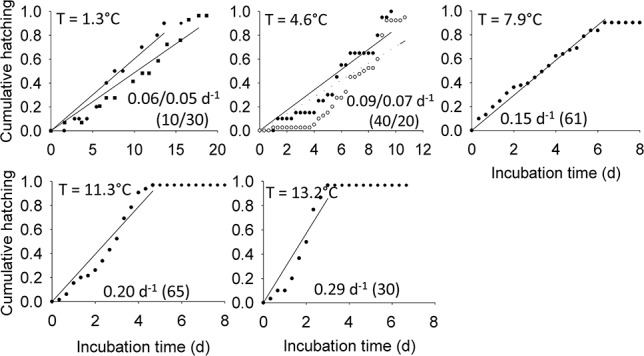
*Oithona similis*. Cumulative hatching (ratio) as a function of temperature (*T*) at five different temperatures, with the lines representing a linear model of its increase over time. The hatching rate (slope of linear model, d^−1^) and number of individuals incubated (*n*) are given for each incubation. Different symbols indicate replicate experiments. Open circles and dashed lines are used for experiments on egg sacs alone, while black circles and squares and full lines are used for replicates of experiments with egg sacs attached to females. Note the different incubation times (*x*-axis).

### Statistics

Data are presented as means with standard deviation (mean ± SD) when available. The effect of temperature on hatching rate (HR), hatching success of clutches (HS_C_), hatching success of eggs within clutches (HS_E_), total hatching success (HS_T_), and development time (D; *M. pusillus* only) was tested using linear regressions following a Shapiro–Wilk normality test. If the assumption of normality was not met, the correlation between two variables was tested by the nonparametric Mann–Whitney rank-sum test. The differences in egg production of *M. pusillus* between incubation temperatures and sampling times were tested using a Kruskal–Wallis 1-way analysis of variance (ANOVA) on Ranks because the dataset could not be normalized due to a high number of zero values. Differences in clutch sizes between temperatures and sampling times were tested by two separate 1-way ANOVAs. These were followed by Holm-Sidak’s *post hoc* test to test for significant differences between groups. All statistical analyses were conducted with SigmaPlot 14.

## RESULTS

### Environmental background

Trends in temperature and Chl *a* followed a typical seasonal succession for Balsfjord. In March, the water column (0–100 m) was homogeneous, with temperatures of ~2°C (Table I) and Chl *a* concentration below detection limits. By May, the surface temperature had increased to 3.5°C, and a thermocline was developing, with a temperature of 2.0°C at 20 m. Chl *a* peaked at 20 m with 1.2 μg L^−1^. In June, the water column was stratified with warmer surface waters (~11.4°C in 2017 and 8.0°C in 2018), dropping to 6.5°C (2017) and 5.3°C (2018) at 20 m, and with a Chl *a* peak of 3.4 μg L^−1^ in 2017 and 1.0 μg L^−1^ in 2018 at 10 m. In August, the water column was still stratified, with warm surface temperatures (9.0°C in 2017 and 10.2°C in 2018) decreasing to 6.8°C at 50 m. The maximum Chl *a* concentration was 0.9 μg L^−1^ (20 m depth). Hence, the copepods collected for incubations in early spring (March) had experienced low temperatures and low Chl *a*. The copepods collected in late spring (May) had been subject to slightly warmer temperatures and increasing Chl *a* concentration, and the copepods collected in early and late summer (June and August) had experienced a combination of a relatively warm surface temperature and medium to high Chl *a* concentrations.

### Egg hatching rate and hatching success

**Fig. 2:**
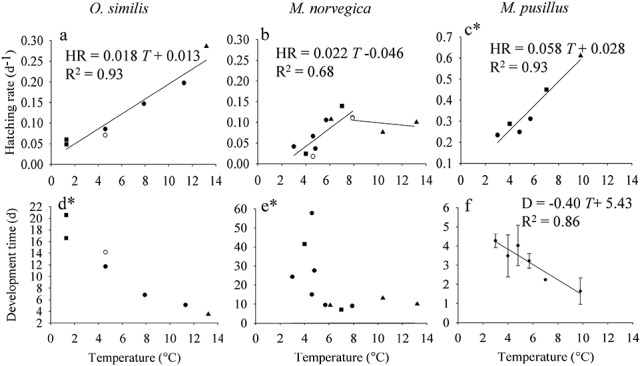
(**a**–**c**) Egg hatching rates (HR, d^−1^) and (**d**–**f**) Development time (D, d) of (a + d) *Oithona similis*, (b + e) *Microsetella norvegica* and (c + f) *Microcalanus pusillus* as a function of temperature. ^*^ The data were calculated as reciprocals of the experimentally obtained values. The seasons are represented by different symbols: squares for spring, circles for early summer, and triangles for late summer. The lines are the linear regressions made from the pooled data including all seasons, with their equations displayed when a linear model was fitting. Note the difference in the hatching rate scale (*y*-axis) for c. Color coding as in Fig. 1.

**Fig. 3:**
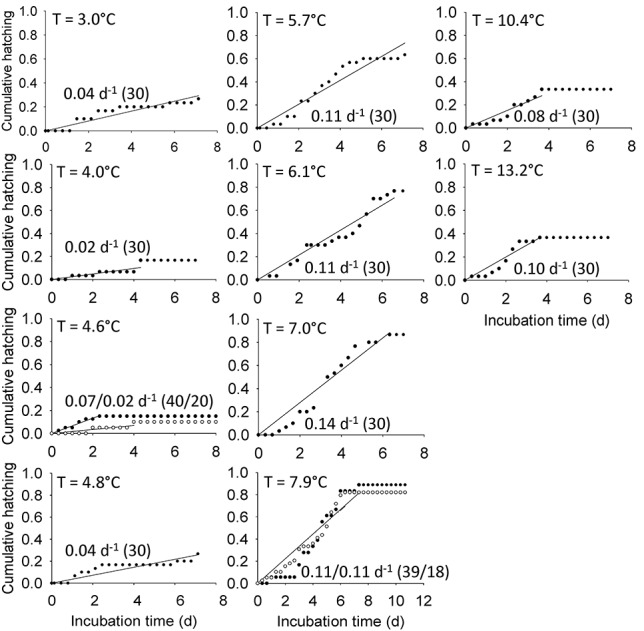
*Microsetella norvegica*. Cumulative hatching (ratio) as a function of temperature at 10 different temperatures, with the lines representing a linear model of its increase over time. The hatching rate (slope of linear model, d^−1^) and number of individuals incubated (*n*) are given for each incubation. Different symbols indicate replicate experiments. Open circles and dashed lines are used for experiments on egg sacs alone, while black circles and full lines are used for replicates of experiments with egg sacs attached to females. Note the different incubation times (*x*-axis).

The egg hatching rate of *O. similis* increased from 0.05–0.06 d^−1^ at 1.3°C to 0.29 d^−1^ at 13.2°C (Fig. 1), and was correlated to temperature (linear regression, *P* < 0.001; Fig. 2a). The egg hatching rate of *M. norvegica* was lowest at temperatures < 4.8°C (<0.07 d^−1^; Fig. 3), reached a maximum of 0.14 d^−1^ at 7.0°C but decreased slightly at temperatures ≥ 7.9°C (0.1 d^−1^, Fig. 2b). *M. norvegica* egg hatching rate was thus positively correlated to temperature within the temperature range 3.0 to 7.9°C (*P* = 0.003). The mean development time of *M. pusillus* eggs decreased from 4.3 ± 0.4 d at 3.0°C to 1.6 ± 0.7 d at 9.8°C, and was linearly correlated to temperature (*P* < 0.01, Fig. 2f). Therefore*, M. pusillus* egg hatching rate, calculated as the reciprocal of the mean development time, increased from 0.23 d^−1^ at 3.0°C to a maximum of 0.61 d^−1^ at 9.8°C (Fig. 2c).

**Fig. 4:**
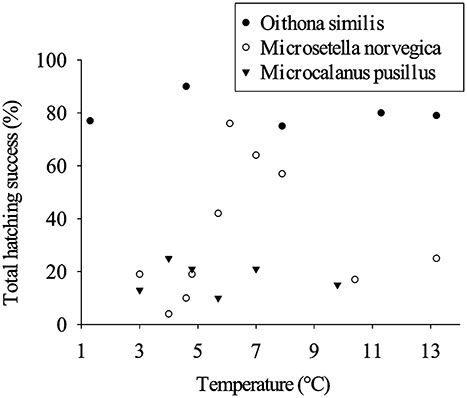
Total hatching success (percentage) of *Oithona similis*, *Microsetella norvegica* and *Microcalanus pusillus* eggs as a function of temperature.

During the 7-days incubations, ≥90% of *O. similis* clutches hatched (HS_C_, Table II). Average egg hatching success within clutches (HS_E_) was between 79 and 93%, and total egg hatching success (HS_T_) varied between 75 and 90% (Fig. 4). *M. norvegica* had a highly variable percentage of clutches that hatched (13–87%; Table II). At low temperatures (<5°C), the average HS_C_ was low (13–37%), while between 66 and 87% of the clutches hatched at temperatures from 5 to 8°C. HS_C_ decreased to 33–37% during late summer at temperatures of >10°C. In total, >50% of the eggs in each clutch hatched (HS_E_, Table II), except for the 4.0°C incubation (May 2018). The combination of *M. norvegica* HS_C_ and HS_E_ resulted in a bell-shaped distribution of the total egg hatching success (HS_T_, Fig. 4): the HS_T_ was ≤25% at the lowest and highest incubation temperatures, but peaked at temperatures between 5 and 8°C. For *M. pusillus*, only 27 to 47% of the clutches hatched (HS_C_) during the 6-days incubations, with 28 to 65% hatching success of the eggs within the clutches (HS_E_, Table II). Therefore, *M. pusillus* total egg hatching success (HS_T_) was ≤25% for all incubations (Fig. 4). None of the variables contributing to egg hatching success (HS_C_, HS_E_ and HS_T_) were correlated to incubation temperature for the three copepod species (linear regressions, all *P* ≥ 0.336).

### Seasonal variations in clutch size, carbon content and specific egg production rate

In our study, *O. similis* females carried eggs from March to late August, whereas *M. norvegica* only started carrying eggs from May onwards. Ovigerous *M. pusillus* females were present throughout the study, and represented 51% ± 9% of the incubated females, independent of temperature (linear regression, *P* = 0.883). Average clutch sizes of *O. similis* and *M. norvegica* peaked in June (Table III), and differed significantly between months (ANOVA on ranks, *P* < 0.001). *O. similis* had larger clutches (~23 ± 9 eggs clutch^−1^) than *M. norvegica* at all seasons (~12 ± 3 eggs clutch^−1^; Table III). The clutch size of *M. pusillus* varied over time (ANOVA on ranks: *P* = 0.003): the ovigerous females produced fewer eggs in June (6 ± 5 eggs female^−1^) than in May (9 ± 3 eggs female^−1^) and August (12 ± 8 eggs female^−1^, Table III). Higher temperatures increased the numbers of eggs produced by ovigerous females but temperature could only explain a small part of the variation in egg production rate of *M. pusillus* (linear regression: *P* = 0.004, *R*^2^ = 0.08).

**Table II:** Range and mean (±SD) of hatching success of clutches (HS_C_, %; based on the first appearance of a freely swimming nauplius) and egg hatching success within clutches (HS_E_, %; mean ± SD) for the three copepod species obtained within all incubations

Species	HS_C_		HS_E_	
	Range	Mean ± SD	Range	Mean ± SD
*Oithona similis*	90–97	94 ± 3	79–93	84 ± 5
*Microsetella norvegica*	13–87	47 ± 28	21–92	66 ± 18
*Microcalanus pusillus*	27–47	36 ± 7	38–65	49 ± 9

Female carbon weight was lowest in February and peaked in June for *O. similis* and *M. pusillus*. The carbon content of *O. similis* females ranged from 0.32 to 0.61 μg C female^−1^ and the carbon content of *M. pusillus* females from 0.76 to 1.26 μg C female^−1^ (Table IV). Female *M. norvegica* carbon weight was lower in April (0.32 μg C female^−1^) than in August (0.51 μg C female^−1^; Table IV). The eggs of *O. similis* and *M. norvegica* were of similar size (diameter of 58 ± 3 and 59 ± 4 μm, respectively), equivalent to a calculated carbon content of 14 × 10^−3^ and 15 × 10^−3^ μg C egg^−1^, respectively. *M. pusillus* eggs were larger (diameter of 65 ± 10 μm) than the sac-spawners’ eggs, and therefore had a higher carbon content (20 × 10^−3^ μg C egg^−1^).

**Fig. 5:**
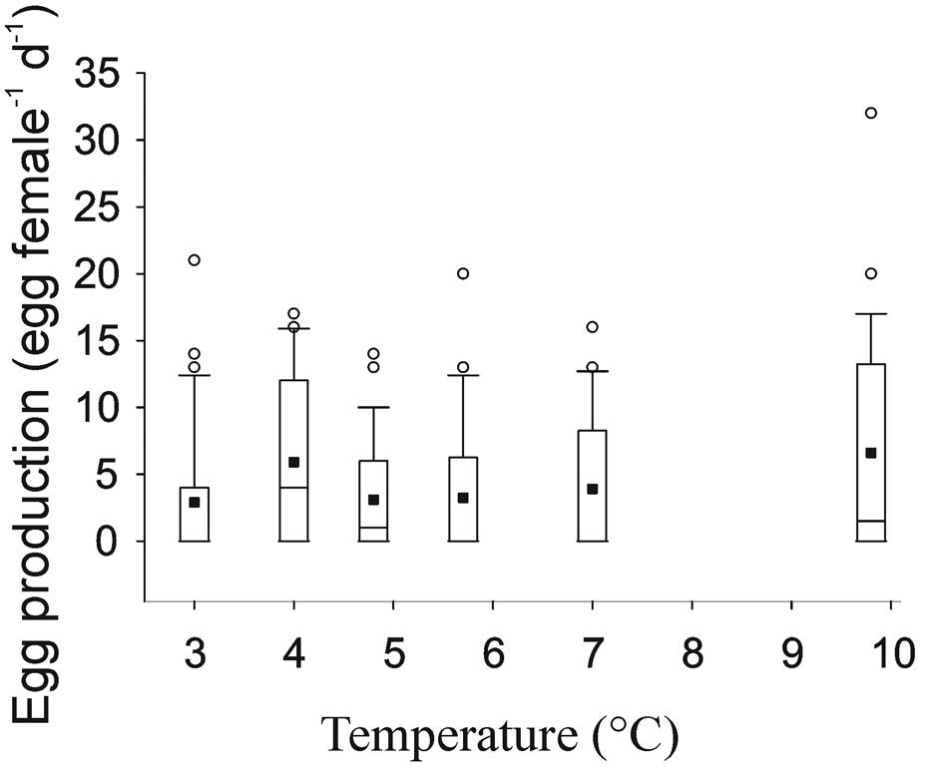
Egg production of *Microcalanus pusillus* as a function of temperature (°C). The bottom and top of the box are the 25th and 75th percentiles with median indicated by a line inside the box (often not visible because it superposes with the 25th percentile). The whiskers show the 10th and 90th percentiles. The outliers are shown by open circles outside the box. The black squares represent the average egg production for all incubations.

The mean egg production of all incubated *M. pusillus* females (i.e. including non-producing females) was stable irrespective of the season or temperature (Fig. 5), varying from 2.9 to 6.6 eggs female^−1^ d^−1^. There was no significant linear correlation between the egg production and temperature (*P* = 0.059), nor were there any significant differences between the incubations conducted at different times (ANOVA on ranks: *P* = 0.208). The SEP_OV_ of *M. pusillus* was 0.20 μg C μg^−1^ C d^−1^ in May, 0.09 μg C μg^−1^ C d^−1^ in June and 0.22 μg C μg^−1^ C d^−1^ in August (Table III), and similar to egg production, independent of temperature.

**Table III:** Mean clutch size (±SD), clutch to female carbon ratio (C_clutch_/C_♀_, %) and specific egg production rate of ovigerous female (SEP_OV_, μg C μg^−1^ C d^−1^) for each experimental date

Species	Date	Clutch size	C_clutch_/C_♀_	SEP_OV_
*Oithona similis*	9.06.2017	20 ± 9	45 ± 20	0.10
	19.06.2017	23 ± 9	51 ± 20	0.10
	15.08.2017	17 ± 7	42 ± 17	0.07
	1.03.2018	8 ± 3	30 ± 11	0.02
	12.03.2018	9 ± 2	39 ± 9	0.02
*Microsetella norvegica*	9.06.2017	11 ± 2	50 ± 9	0.06
	19.06.2017	10 ± 3	45 ± 14	0.05
	15.08.2017	9 ± 3	33 ± 11	0.03
	3.05.2018	10 ± 1	58 ± 6	0.03
	11.06.2018	12 ± 3	54 ± 14	0.05
	24.08.2018	9 ± 2	33 ± 7	0.03
*Microcalanus pusillus*	12.03.2018	Spawning observed but not quantified		
	3.05.2018	9 ± 3	20 ± 7	0.20
	11.06.2018	6 ± 5	9 ± 8	0.09
	24.08.2018	12 ± 8	22 ± 15	0.22

Clutch sizes are pooled from all experiments started the same day, and the SEP_OV_ is calculated based on the mean clutch size and surface water temperature

The SEP_OV_ of *O. similis* was lowest in spring at 0.02 μg C μg^−1^ C d^−1^ (Table III), peaked in June at a maximum of 0.12 μg C μg^−1^ C d^−1^, thereafter decreasing to 0.07 μg C μg^−1^ C d^−1^ in late summer. *O. similis* SEP_OV_ was correlated to surface temperatures (linear regression, *P* = 0.004). For *M. norvegica*, the SEP_OV_ was relatively low and ranged from 0.03 to 0.06 μg C μg^−1^ C d^−1^ (Table III), with the highest values during the summer. In contrast to *O. similis*, the SEP_OV_ of *M. norvegica* was independent of the surface temperatures (linear regressions; *P* > 0.05).

### *Microcalanus* species identification

The genetic identification of the 58 *Microcalanus* females used in the incubations revealed that only *M. pusillus* were present (Table SI). The morphological examination of the specimens matched the genetic species identification as only one morphological type of *Microcalanus* was observed, with short antennae. Therefore, we assume that all *Microcalanus* in our incubations were *M. pusillus*.

### Discussion

Egg production, hatching success and egg development time differed between the three species, as did their response to temperature. *O. similis* and *M. pusillus* had increasing egg hatching rates over the full temperature range studied, with a shorter development time but lower hatching success for the broadcast spawner *M. pusillus*. In contrast, *M. norvegica* had maximum egg hatching rate at 8°C, and a decrease thereafter. *M. norvegica* also had the lowest specific egg production at all time-points. It appears that *O. similis* and *M. pusillus* could increase their reproductive output with increasing temperature, whereas *M. norvegica* was most productive between 6 and 8°C. The observed differences could neither be attributed to body size for these similar-sized species, nor to their reproductive strategy (sac spawners versus free spawner). Our study demonstrates that small copepod species show variable responses of egg hatching and productivity to temperature.

**Table IV:** Mean sizes (±SD, μm) and carbon weight of female copepods (C_♀_, μg C) by date

Species	Date	Female size	C_♀_
*Oithona similis*	23.08.2016	472 ± 48	0.54
	27.02.2017	429 ± 25	0.36
	17.03.2017	490 ± 16	0.32
	7.04.2017	440 ± 36	0.48
	11.06.2018		0.61
*Microsetella norvegica*	23.08.2016	463 ± 24	0.51
	7.04.2017	471 ± 16	0.32
	11.06.2018	478 ± 22	0.42^a^
*Microcalanus pusillus*	23.08.2016	494 ± 48	1.05^b^
	27.02.2017	450 ± 21	0.76
	7.04.2017	521 ± 34	0.87
	11.06.2018	539 ± 36	1.26

^a^Due to technical problems, the true carbon value was lost and it is approximated as the average between the female carbon weights of April and August.

^b^The carbon value may be underestimated as the filter contained some stage five copepodites due to the scarcity of females.

### Temperature dependence of hatching rates and hatching success

The threefold increase of the egg hatching rate of the broadcast-spawning *M. pusillus*, within the 10°C increase in temperature, is comparable to that of the small calanoid *Pseudocalanus* spp. that tripled its hatching rate between 1 and 7°C (Middelbo *et al.,* 2019). In a previous study, a Q_10_ of 2.45 was found for the egg hatching rate of broadcast spawners (Hirst and Bunker, 2003), which is comparable to our findings. *M. pusillus* is a sub-surface species (Norrbin, 1991), and is mostly found <50 m in Balsfjord where water masses were <6°C during the study. Previously, this species has probably been grouped with *M. pygmaeus* as *Microcalanus* spp. (Hop *et al.,* 2019b; Madsen *et al.,* 2008; Riisgaard *et al.,* 2014; Walkusz *et al.,* 2009), which was observed at temperatures within the range from −2 to 13°C in the Arctic. In our study, the egg hatching rate of *M. pusillus* showed a strong response to increasing temperature, suggesting that its reproductive rate would increase with an increase in temperature. Similar to the typically high egg hatching rates of other broadcast spawners (Hirst and Bunker, 2003; Mauchline, 1998), the non-motile free-floating eggs of *M. pusillus* hatch rapidly, perhaps easing the risk of cannibalism and predation on the eggs (Hirst and Lopez-Urrutia, 2006; Kiørboe and Sabatini, 1994; Weydmann *et al.,* 2015). Higher temperatures could thus increase early-stage survival of *M. pusillus* by ensuring a faster transition from a non-motile to a motile stage.

In contrast to the short egg hatching time of broadcast-spawning copepods, a longer egg development time is characteristic of egg-carrying copepods (Kiørboe and Sabatini, 1994). An egg-carrying strategy usually constrains lifetime fecundity. It may prove disadvantageous in cases of high mortality of egg-bearing females (Ward and Hirst, 2007), but will pay off in environments where predation is high on pelagic eggs (Kiørboe and Sabatini, 1994). An increase in temperature might change the cost–benefit ratio of the egg-carrying strategy if mortality and egg hatching time respond differently to increasing temperature. This could be the case for *M. norvegica* where the maximum egg hatching rate was reached at 7°C with no further increase at higher temperatures, which describes a performance curve. Performance curves, i.e. the curve illustrating the effect of a climatic variable like temperature on a physiological rate, are often bell-shaped (Dam and Baumann, 2018). However, previous studies on copepods have reported a positive linear or power relationship between investigated temperatures and egg development times (e.g. Andersen and Nielsen, 1997, Ianora *et al.,* 2007, Mclaren, 1966, Middelbo *et al.,* 2019, Nielsen *et al.,* 2002, our observations of *O. similis*). The performance curve of *M. norvegica* hatching rate was therefore surprising. However, it is probable that an optimum may be reached for any particular physiological rate, given that the range of the climatic variable (e.g. temperature) is large enough for that species.

It is possible that the bell-shaped temperature response of *M. norvegica* egg hatching rate reflected an adaptation to temperatures that prevail during the reproductive season. The egg hatching rate of copepods reflects development of an egg, as a reciprocal measure of the time spent between two developmental stages (Huntley and Lopez, 1992). Development and growth can indeed reach a maximum, after which growth may not further increase with increasing temperature or can be negatively affected (Lee *et al.,* 2003; Lonsdale and Levinton, 1986). It is possible that northern harpacticoid populations, including *M. norvegica*, reach their maximum growth and egg hatching rate at lower temperatures than southern populations, as a local adaptation to the prevailing temperatures. In Balsfjord, egg-carrying females *M. norvegica* are found between May and September (Svensen *et al.,* 2018), mostly above 50 m where temperatures are usually between 5 and 8°C (Eilertsen and Skarðhamar, 2006). Therefore, the Balsfjord population may have adapted to the local temperatures. *M. norvegica* and *O. similis* had comparable egg hatching rate only between 6 and 8°C, whereas the egg hatching rate of *O. similis* surpassed that of *M. norvegica* >8°C. Hence, *O. similis* appeared more thermally plastic than *M. norvegica.* The egg hatching rates of *O. similis* were similar to those observed by Nielsen *et al.* (2002).

In the Arctic, numerical dominance of *M. norvegica* seems to be confined to fjords (Arendt *et al.,* 2013; Hjorth and Dahllöf, 2008; Svensen *et al.,* 2018), whereas *O. similis* can be abundant both in fjords (Hop *et al.,* 2019b; Middelbo *et al.,* 2019) and coastal and shelf areas (Dvoretsky and Dvoretsky, 2009a; Dvoretsky and Dvoretsky, 2015; Hop *et al.,* 2019a; Madsen *et al.,* 2008). *M. norvegica* is present from tropical seas with temperatures > 30°C (Chew and Chong, 2016) to polar areas (Arendt *et al.,* 2013; Svensen *et al.,* 2019). In the Inland Sea of Japan, the population of *M. norvegica* did not reach a maximum egg hatching rate within the temperature investigated (Uye *et al.,* 2002), an observation that differs from ours. This suggests that although *M. norvegica* is present in widely different environments, populations may adapt to local conditions. For *M. norvegica* in Balsfjord, this could mean that recruitment of *M. norvegica* may decline if the temperature increases >8°C during the reproductive period, due to their lowered egg hatching rates at higher temperatures. In contrast, *O. similis* and *M. pusillus* may benefit as a higher temperature increased their egg hatching rate. A higher thermal plasticity of *O. similis* and *M. pusillus* suggests a higher recruitment potential than for *M. norvegica* (Allan, 1976; Devreker *et al.,* 2012; Tang *et al.,* 1998) in Balsfjord, although other processes linked to mortality and survival will also be important to shape the recruitment of species.

Other than the thermal plasticity of the egg hatching rate, the recruitment potential of a species is affected by its egg hatching success (Devreker *et al.,* 2012). Hatching success can be influenced by temperature (Hansen *et al.,* 2010), excreted substances from phytoplankton (Ambler, 1985; Ianora *et al.,* 2007), successful mating/fertilization (Mironova and Pasternak, 2017), and food composition (Jónasdóttir *et al.,* 2005). We found no correlation between egg hatching success and temperature or season, but notable differences were observed between species. *O. similis* had a high total egg hatching success compared to *M. norvegica* and *M. pusillus.* Though the egg hatching success of *M. norvegica* was not linearly related to temperature, the highest egg hatching success was found at the optimum temperatures for the egg hatching rate. This agrees with the possibility of a local temperature adaptation of *M. norvegica*.

The low egg hatching success of *M. pusillus* contrasted with the high thermal plasticity of its egg hatching rate. Egg hatching success of calanoid copepod eggs is rarely <60% (e.g. Devreker *et al.,* 2012; Hansen *et al.,* 2010; Tang *et al.,* 1998), although episodically low hatching success (0–30%) has been observed (e.g. Halsband-Lenk *et al.,* 2001; Ianora and Poulet, 1993; Jónasdóttir *et al.,* 2005; Miralto *et al.,* 1998; Yamaguchi *et al.,* 2010). To our knowledge, no previous estimates of egg hatching success exist for *M. pusillus*. In our incubations, most of the *M. pusillus* eggs that did not hatch were discolored or disintegrated, and only ~4% of the unhatched eggs seemed still viable at the end of the 6 days observation. Therefore, we assume that the incubated *M. pusillus* female produced mainly subitaneous eggs (i.e. eggs hatching without delay), and that the low egg hatching success was representative of the *in situ* conditions. It should be noted that the incubation methods used in our study followed established methods for broadcast-spawning copepods (Drillet *et al.,* 2008; Halvorsen, 2015), and female mortality was low. A low *in situ* hatching success, as observed in our study, would lower the positive effect of a temperature increase on the egg hatching rate and thereby on the recruitment potential of *M. pusillus*.

### Seasonality

Egg-producing females were present at least from March to August (*O. similis* and *M. pusillus*) and from May to August (*M. norvegica*) in Balsfjord, which is in accordance with the long reproductive periods described in previous studies (Dvoretsky and Dvoretsky, 2009b; Norrbin, 1991; Svensen *et al.,* 2018). Small copepods do not accumulate large lipid reserves (Arima *et al.,* 2014; Norrbin, 1991), contrasting with larger diapausing, and sometimes capital-breeding, copepods (Conover, 1988; Sainmont *et al.,* 2014; Varpe *et al.,* 2009). Smaller copepod species typically rely on continuous feeding to fuel their reproduction (Norrbin, 1991; Svensen *et al.,* 2019); i.e. income breeding. The three investigated species are omnivorous, grazing on food sources often available outside the spring-bloom period, such as marine aggregates (Koski *et al.,* 2005; Norrbin, 1991) and microzooplankton (Castellani *et al.,* 2005; Svensen and Kiørboe, 2000).

The egg production rate for ovigerous females (SEP_OV_) of *M. norvegica* was unusually low for a sac spawner at all seasons (Uye and Sano, 1995). In temperate waters, *M. norvegica* carried 15.8 eggs female^−1^ with an egg hatching rate of 0.67 d^−1^ at 27.8°C (Uye *et al.,* 2002). Based on Equation 1, the weight-specific egg production rate for the egg-bearing females in the Inland Sea of Japan may be as high as 0.34 μg C μg^−1^ C d^−1^, which demonstrates a high production potential of *M. norvegica* at high temperature. The difference in egg production rates between *O. similis* and *M. norvegica* in Balsfjord may reflect diverse reproductive investments. Even if the two species carry eggs, the time spent carrying eggs may differ*.* Female *M. norvegica* are suggested to have a hybrid egg-carrying strategy, where they release their egg sac before the eggs have hatched (Koski *et al.,* 2014). In that case, the egg hatching rate may not represent the time interval between two clutches, resulting in a potential underestimation of *M. norvegica* egg production rate.

We observed that *M. pusillus* had a SEP_OV_ ~3.1 times higher than *O. similis*, and ~7.3 times higher SEP_OV_ than *M. norvegica*. Broadcast spawners have on average a 2.5 times higher weight-specific egg production rate than sac spawners, to compensate for high egg mortality (Kiørboe and Sabatini, 1995). However, we found that the difference in SEP_OV_ between species varied with seasons. Adverse environmental conditions may cause physiological stress, which could lower the egg production of copepods (Uye and Sano, 1995). *M. pusillus* egg production peaked in May and August and the sac spawners had a peak SEP_OV_ in June. The differences between months were significant but not related to surface temperature (except for *O. similis*). The SEP_OV_ is influenced by the egg hatching rate, clutch size and female body weight (Equation 1 and 2). Egg hatching rates investigated at similar temperatures but different months showed no significant differences. Therefore, the seasonal variation observed likely resulted from the changes in the clutch size and the female body weight (i.e. female condition). Food availability and quality varies between March and August in Balsfjord (Eilertsen *et al.,* 1981), which can affect clutch size (Ambler, 1985; Castellani *et al.,* 2007; Halsband and Hirche, 2001) and carbon weight of copepods (Auel and Hagen, 2005, this study). The seasonal pattern in the weight-specific egg production rate of the three copepod species is likely the result of seasonal variation in abiotic and biotic factors that influence clutch size and female weight along with the temperature-dependency of the egg hatching rate.

## CONCLUSION

In this study, we provide egg hatching rate and egg hatching success data for three small and abundant copepod species. The egg hatching rates of all three species responded to increasing temperatures but their thermal plasticity differed. Our study therefore highlights species-specific temperature dependencies also within the abundant group of small copepods. Supporting previous observations, we confirmed that small sub-arctic broadcast spawners have faster egg development than co-occurring sac spawners and that their weight-specific egg production rate is higher. Moreover, we found that the weight-specific egg production of ovigerous females varies seasonally, presumably influenced by the seasonal changes in the clutch size and carbon content of the female of the three species. This study therefore also highlights the importance of documenting vital rates at different seasons. In the future, oceans will have conditions combining new ranges of temperature, salinity, pH, oxygen and primary production (IPCC, 2019), including changes in the seasonality of these variables. According to our findings, the consequences of these new conditions will differ across species and potentially impact their phenology and relative biomass. Such alterations may in turn interact with the predator–prey interactions or the cycling of organic matter in the pelagic realm, both of which have implications for the energy flux and carbon turnover.

## Supplementary Material

Supplementary_material_Table_SI_fbaa039
