# Supplementary material for: Temperature-dependent egg production and egg hatching rates of small egg-carrying and broadcast-spawning copepods Oithona similis, Microsetella norvegica and Microcalanus pusillus
Source: J Plankton Res. 2020 Sep 7;42(5):564–80. doi: 10.1093/plankt/fbaa039 (PMC7484935; doi:10.1093/plankt/fbaa039)
Supplement: Supplementary_material_Table_SI_fbaa039 [file supplementary_material_table_si_fbaa039.pdf]

Table SI. DNA sequences of the Leray fragment of the COI for the 58 Microcalanus *females and best match for the sequence on BOLD.*

Sample #	Best identity	Species name	Best match	Sequence length	Sequence
1	1	Microcalanus pusillus	CAISN 950-13	313	TCTTTCAAGAAATATTGCACACGCAGGGGGCTCAGTAGA TTTTGCTATTTTTTCTCTACATTTGGCCGGGGTGAGATCA ATTTTAGGGGCAGTTAATTTTATTAGGACTTTAGGAAACC TGCGAGTCTTTGGTATATTACTGGACCGGATACCATTATT CGCATGGTCTGTCTTAGTCACTGCGGTGCTGCTATTGCTC TCTATGCCAGTACTCGCTGGCGCGATTACTATGCTTCTAA CAGACCGAAATTTAAACACGTCGTTTTATGATGTGGGAG GGGGTGGAGACCCTATCTTATACCAACATCTTTTT
2	0.996805112	Microcalanus pusillus	CAISN 950-13	313	TCTTTCAAGAAATATTGCACACGCAGGGGGCTCAGTAGA TTTTGCTATTTTTTCTCTACATTTGGCCGGGGTGAGATCA ATTTTAGGGGCAGTTAATTTTATTAGGACTTTAGGAAACC TGCGAGTCTTTGGCATATTACTGGACCGGATACCATTATT CGCATGGTCTGTCTTAGTCACTGCGGTGCTGCTATTGCTC TCTATGCCAGTACTCGCTGGCGCGATTACTATGCTTCTAA CAGACCGAAATTTAAACACGTCGTTTTATGATGTGGGAG GGGGTGGAGACCCTATCTTATACCAACATCTTTTT
3	0.996805112	Microcalanus pusillus	CAISN 950-13	313	TCTTTCAAGAAATATTGCACACGCAGGGGGCTCAGTAGA TTTTGCTATTTTTTCTCTACATTTGGCCGGGGTGAGATCA ATTTTAGGGGCAGTTAATTTTATTAGGACTTTAGGAAACC TGCGAGTCTTTGGTATATTACTGGACCGGATACCATTATT CGCATGGTCTGTCTTAGTCACTGCGGTGCTGCTATTGCTC TCTATGCCAGTACTCGCTGGTGCGATTACTATGCTTCTAA CAGACCGAAATTTAAACACGTCGTTTTATGATGTGGGAG GGGGTGGAGACCCTATCTTATACCAACATCTTTTT

Sample #	Best identity	Species name	Best match	Sequence length	Sequence
4	1	Microcalanus pusillus	CAISN 950-13	313	TCTTTCAAGAAATATTGCACACGCAGGGGGCTCAGTAGATTTTGCTATTTTTTCTCTACATTTGGCCGGGGTGAGATCAATTTTAGGGGCAGTTAATTTTATTAGGACTTTAGGAAACCTGCGAGTCTTTGGTATATTACTGGACCGGATACCATTATTCGCATGGTCTGTCTTAGTCACTGCGGTGCTGCTATTGCTCTCTATGCCAGTACTCGCTGGCGCGATTACTATGCTTCTAACAGACCGAAATTTAAACACGTCGTTTTATGATGTGGGAGGGGGTGGAGACCCTATCTTATACCAACATCTTTTT
5	1	Microcalanus pusillus	CAISN 950-13	313	TCTTTCAAGAAATATTGCACACGCAGGGGGCTCAGTAGATTTTGCTATTTTTTCTCTACATTTGGCCGGGGTGAGATCAATTTTAGGGGCAGTTAATTTTATTAGGACTTTAGGAAACCTGCGAGTCTTTGGTATATTACTGGACCGGATACCATTATTCGCATGGTCTGTCTTAGTCACTGCGGTGCTGCTATTGCTCTCTATGCCAGTACTCGCTGGCGCGATTACTATGCTTCTAACAGACCGAAATTTAAACACGTCGTTTTATGATGTGGGAGGGGGTGGAGACCCTATCTTATACCAACATCTTTTT
6	1	Microcalanus pusillus	CAISN 950-13	313	TCTTTCAAGAAATATTGCACACGCAGGGGGCTCAGTAGATTTTGCTATTTTTTCTCTACATTTGGCCGGGGTGAGATCAATTTTAGGGGCAGTTAATTTTATTAGGACTTTAGGAAACCTGCGAGTCTTTGGTATATTACTGGACCGGATACCATTATTCGCATGGTCTGTCTTAGTCACTGCGGTGCTGCTATTGCTCTCTATGCCAGTACTCGCTGGCGCGATTACTATGCTTCTAACAGACCGAAATTTAAACACGTCGTTTTATGATGTGGGAGGGGGTGGAGACCCTATCTTATACCAACATCTTTTT
7	1	Microcalanus pusillus	CAISN 950-13	313	TCTTTCAAGAAATATTGCACACGCAGGGGGCTCAGTAGATTTTGCTATTTTTTCTCTACATTTGGCCGGGGTGAGATCAATTTTAGGGGCAGTTAATTTTATTAGGACTTTAGGAAACCTGCGAGTCTTTGGTATATTACTGGACCGGATACCATTATTCGCATGGTCTGTCTTAGTCACTGCGGTGCTGCTATTGCTCTCTATGCCAGTACTCGCTGGCGCGATTACTATGCTTCTAACAGACCGAAATTTAAACACGTCGTTTTATGATGTGGGAGGGGGTGGAGACCCTATCTTATACCAACATCTTTTT

Sample #	Best identity	Species name	Best match	Sequence length	Sequence
8	1	Microcalanus pusillus	CAISN 950-13	313	TCTTTCAAGAAATATTGCACACGCAGGGGGCTCAGTAGATTTTGCTATTTTTTCTCTACATTTGGCCGGGGTGAGATCAATTTTAGGGGCAGTTAATTTTATTAGGACTTTAGGAAACCTGCGAGTCTTTGGTATATTACTGGACCGGATACCATTATTCGCATGGTCTGTCTTAGTCACTGCGGTGCTGCTATTGCTCTCTATGCCAGTACTCGCTGGCGCGATTACTATGCTTCTAACAGACCGAAATTTAAACACGTCGTTTTATGATGTGGGAGGGGGTGGAGACCCTATCTTATACCAACATCTTTTT
9	1	Microcalanus pusillus	CAISN 950-13	313	TCTTTCAAGAAATATTGCACACGCAGGGGGCTCAGTAGATTTTGCTATTTTTTCTCTACATTTGGCCGGGGTGAGATCAATTTTAGGGGCAGTTAATTTTATTAGGACTTTAGGAAACCTGCGAGTCTTTGGTATATTACTGGACCGGATACCATTATTCGCATGGTCTGTCTTAGTCACTGCGGTGCTGCTATTGCTCTCTATGCCAGTACTCGCTGGCGCGATTACTATGCTTCTAACAGACCGAAATTTAAACACGTCGTTTTATGATGTGGGAGGGGGTGGAGACCCTATCTTATACCAACATCTTTTT
10	1	Microcalanus pusillus	CAISN 950-13	313	TCTTTCAAGAAATATTGCACACGCAGGGGGCTCAGTAGATTTTGCTATTTTTTCTCTACATTTGGCCGGGGTGAGATCAATTTTAGGGGCAGTTAATTTTATTAGGACTTTAGGAAACCTGCGAGTCTTTGGTATATTACTGGACCGGATACCATTATTCGCATGGTCTGTCTTAGTCACTGCGGTGCTGCTATTGCTCTCTATGCCAGTACTCGCTGGCGCGATTACTATGCTTCTAACAGACCGAAATTTAAACACGTCGTTTTATGATGTGGGAGGGGGTGGAGACCCTATCTTATACCAACATCTTTTT

Sample #	Best identity	Species name	Best match	Sequence length	Sequence
11	1	Microcalanus pusillus	CAISN 950-13	313	TCTTTCAAGAAATATTGCACACGCAGGGGGCTCAGTAGATTTTGCTATTTTTTCTCTACATTTGGCCGGGGTGAGATCAATTTTAGGGGCAGTTAATTTTATTAGGACTTTAGGAAACCTGCGAGTCTTTGGTATATTACTGGACCGGATACCATTATTCGCATGGTCTGTCTTAGTCACTGCGGTGCTGCTATTGCTCTCTATGCCAGTACTCGCTGGCGCGATTACTATGCTTCTAACAGACCGAAATTTAAACACGTCGTTTTATGATGTGGGAGGGGGTGGAGACCCTATCTTATACCAACATCTTTTT
12	1	Microcalanus pusillus	CAISN 950-13	313	TCTTTCAAGAAATATTGCACACGCAGGGGGCTCAGTAGATTTTGCTATTTTTTCTCTACATTTGGCCGGGGTGAGATCAATTTTAGGGGCAGTTAATTTTATTAGGACTTTAGGAAACCTGCGAGTCTTTGGTATATTACTGGACCGGATACCATTATTCGCATGGTCTGTCTTAGTCACTGCGGTGCTGCTATTGCTCTCTATGCCAGTACTCGCTGGCGCGATTACTATGCTTCTAACAGACCGAAATTTAAACACGTCGTTTTATGATGTGGGAGGGGGTGGAGACCCTATCTTATACCAACATCTTTTT
13	1	Microcalanus pusillus	CAISN 950-13	313	TCTTTCAAGAAATATTGCACACGCAGGGGGCTCAGTAGATTTTGCTATTTTTTCTCTACATTTGGCCGGGGTGAGATCAATTTTAGGGGCAGTTAATTTTATTAGGACTTTAGGAAACCTGCGAGTCTTTGGTATATTACTGGACCGGATACCATTATTCGCATGGTCTGTCTTAGTCACTGCGGTGCTGCTATTGCTCTCTATGCCAGTACTCGCTGGCGCGATTACTATGCTTCTAACAGACCGAAATTTAAACACGTCGTTTTATGATGTGGGAGGGGGTGGAGACCCTATCTTATACCAACATCTTTTT

Sample #	Best identity	Species name	Best match	Sequence length	Sequence
14	1	Microcalanus pusillus	CAISN 950-13	313	TCTTTCAAGAAATATTGCACACGCAGGGGGCTCAGTAGATTTTGCTATTTTTTCTCTACATTTGGCCGGGGTGAGATCAATTTTAGGGGCAGTTAATTTTATTAGGACTTTAGGAAACCTGCGAGTCTTTGGTATATTACTGGACCGGATACCATTATTCGCATGGTCTGTCTTAGTCACTGCGGTGCTGCTATTGCTCTCTATGCCAGTACTCGCTGGCGCGATTACTATGCTTCTAACAGACCGAAATTTAAACACGTCGTTTTATGATGTGGGAGGGGGTGGAGACCCTATCTTATACCAACATCTTTTT
15	1	Microcalanus pusillus	CAISN 950-13	313	TCTTTCAAGAAATATTGCACACGCAGGGGGCTCAGTAGATTTTGCTATTTTTTCTCTACATTTGGCCGGGGTGAGATCAATTTTAGGGGCAGTTAATTTTATTAGGACTTTAGGAAACCTGCGAGTCTTTGGTATATTACTGGACCGGATACCATTATTCGCATGGTCTGTCTTAGTCACTGCGGTGCTGCTATTGCTCTCTATGCCAGTACTCGCTGGCGCGATTACTATGCTTCTAACAGACCGAAATTTAAACACGTCGTTTTATGATGTGGGAGGGGGTGGAGACCCTATCTTATACCAACATCTTTTT
16	1	Microcalanus pusillus	CAISN 950-13	313	TCTTTCAAGAAATATTGCACACGCAGGGGGCTCAGTAGATTTTGCTATTTTTTCTCTACATTTGGCCGGGGTGAGATCAATTTTAGGGGCAGTTAATTTTATTAGGACTTTAGGAAACCTGCGAGTCTTTGGTATATTACTGGACCGGATACCATTATTCGCATGGTCTGTCTTAGTCACTGCGGTGCTGCTATTGCTCTCTATGCCAGTACTCGCTGGCGCGATTACTATGCTTCTAACAGACCGAAATTTAAACACGTCGTTTTATGATGTGGGAGGGGGTGGAGACCCTATCTTATACCAACATCTTTTT

Sample #	Best identity	Species name	Best match	Sequence length	Sequence
17	0.996805112	Microcalanus pusillus	CAISN 950-13	313	TCTTTCAAGAAATATTGCACACGCAGGGGGCTCAGTAGA<br>TTTTGCTATTTTTTCTCTACATTTGGCCGGGGTGAGATCA<br>ATTTTAGGGGCAGTTAATTTTATTAGGACTTTAGGAAACC<br>TGCGAGTCTTTGGTATATTACTGGACCGGATACCATTATT<br>CGCATGGTCTGTCTTAGTCACTGCGGTGCTGCTATTGCTC<br>TCTATGCCAGTACTCGCTGGTGCGATTACTATGCTTCTAA<br>CAGACCGAAATTTAAACACGTCGTTTTATGATGTGGGAG<br>GGGGTGGAGACCCTATCTTATACCAACATCTTTTT
18	1	Microcalanus pusillus	CAISN 950-13	313	TCTTTCAAGAAATATTGCACACGCAGGGGGCTCAGTAGA<br>TTTTGCTATTTTTTCTCTACATTTGGCCGGGGTGAGATCA<br>ATTTTAGGGGCAGTTAATTTTATTAGGACTTTAGGAAACC<br>TGCGAGTCTTTGGTATATTACTGGACCGGATACCATTATT<br>CGCATGGTCTGTCTTAGTCACTGCGGTGCTGCTATTGCTC<br>TCTATGCCAGTACTCGCTGGCGCGATTACTATGCTTCTAA<br>CAGACCGAAATTTAAACACGTCGTTTTATGATGTGGGAG<br>GGGGTGGAGACCCTATCTTATACCAACATCTTTTT
19	1	Microcalanus pusillus	CAISN 950-13	313	TCTTTCAAGAAATATTGCACACGCAGGGGGCTCAGTAGA<br>TTTTGCTATTTTTTCTCTACATTTGGCCGGGGTGAGATCA<br>ATTTTAGGGGCAGTTAATTTTATTAGGACTTTAGGAAACC<br>TGCGAGTCTTTGGTATATTACTGGACCGGATACCATTATT<br>CGCATGGTCTGTCTTAGTCACTGCGGTGCTGCTATTGCTC<br>TCTATGCCAGTACTCGCTGGCGCGATTACTATGCTTCTAA<br>CAGACCGAAATTTAAACACGTCGTTTTATGATGTGGGAG<br>GGGGTGGAGACCCTATCTTATACCAACATCTTTTT

Sample #	Best identity	Species name	Best match	Sequence length	Sequence
20	1	Microcalanus pusillus	CAISN 950-13	313	TCTTTCAAGAAATATTGCACACGCAGGGGGCTCAGTAGATTTTGCTATTTTTTCTCTACATTTGGCCGGGGTGAGATCAATTTTAGGGGCAGTTAATTTTATTAGGACTTTAGGAAACCTGCGAGTCTTTGGTATATTACTGGACCGGATACCATTATTCGCATGGTCTGTCTTAGTCACTGCGGTGCTGCTATTGCTCTCTATGCCAGTACTCGCTGGCGCGATTACTATGCTTCTAACAGACCGAAATTTAAACACGTCGTTTTATGATGTGGGAGGGGGTGGAGACCCTATCTTATACCAACATCTTTTT
21	1	Microcalanus pusillus	CAISN 950-13	313	TCTTTCAAGAAATATTGCACACGCAGGGGGCTCAGTAGATTTTGCTATTTTTTCTCTACATTTGGCCGGGGTGAGATCAATTTTAGGGGCAGTTAATTTTATTAGGACTTTAGGAAACCTGCGAGTCTTTGGTATATTACTGGACCGGATACCATTATTCGCATGGTCTGTCTTAGTCACTGCGGTGCTGCTATTGCTCTCTATGCCAGTACTCGCTGGCGCGATTACTATGCTTCTAACAGACCGAAATTTAAACACGTCGTTTTATGATGTGGGAGGGGGTGGAGACCCTATCTTATACCAACATCTTTTT
22	1	Microcalanus pusillus	CAISN 950-13	313	TCTTTCAAGAAATATTGCACACGCAGGGGGCTCAGTAGATTTTGCTATTTTTTCTCTACATTTGGCCGGGGTGAGATCAATTTTAGGGGCAGTTAATTTTATTAGGACTTTAGGAAACCTGCGAGTCTTTGGTATATTACTGGACCGGATACCATTATTCGCATGGTCTGTCTTAGTCACTGCGGTGCTGCTATTGCTCTCTATGCCAGTACTCGCTGGCGCGATTACTATGCTTCTAACAGACCGAAATTTAAACACGTCGTTTTATGATGTGGGAGGGGGTGGAGACCCTATCTTATACCAACATCTTTTT

Sample #	Best identity	Species name	Best match	Sequence length	Sequence
23	1	Microcalanus pusillus	CAISN 950-13	313	TCTTTCAAGAAATATTGCACACGCAGGGGGCTCAGTAGATTTTGCTATTTTTTCTCTACATTTGGCCGGGGTGAGATCAATTTTAGGGGCAGTTAATTTTATTAGGACTTTAGGAAACCTGCGAGTCTTTGGTATATTACTGGACCGGATACCATTATTCGCATGGTCTGTCTTAGTCACTGCGGTGCTGCTATTGCTCTCTATGCCAGTACTCGCTGGCGCGATTACTATGCTTCTAACAGACCGAAATTTAAACACGTCGTTTTATGATGTGGGAGGGGGTGGAGACCCTATCTTATACCAACATCTTTTT
24	1	Microcalanus pusillus	CAISN 950-13	313	TCTTTCAAGAAATATTGCACACGCAGGGGGCTCAGTAGATTTTGCTATTTTTTCTCTACATTTGGCCGGGGTGAGATCAATTTTAGGGGCAGTTAATTTTATTAGGACTTTAGGAAACCTGCGAGTCTTTGGTATATTACTGGACCGGATACCATTATTCGCATGGTCTGTCTTAGTCACTGCGGTGCTGCTATTGCTCTCTATGCCAGTACTCGCTGGCGCGATTACTATGCTTCTAACAGACCGAAATTTAAACACGTCGTTTTATGATGTGGGAGGGGGTGGAGACCCTATCTTATACCAACATCTTTTT
25	0.996805112	Microcalanus pusillus	CAISN 950-13	313	TCTTTCAAGAAATATTGCACACGCAGGGGGCTCAGTAGATTTTGCTATTTTTTCTCTACATTTGGCCGGGGTGAGATCAATTTTAGGGGCAGTTAATTTTATTAGGACTTTAGGAAACCTGCGAGTCTTTGGCATATTACTGGACCGGATACCATTATTCGCATGGTCTGTCTTAGTCACTGCGGTGCTGCTATTGCTCTCTATGCCAGTACTCGCTGGCGCGATTACTATGCTTCTAACAGACCGAAATTTAAACACGTCGTTTTATGATGTGGGAGGGGGTGGAGACCCTATCTTATACCAACATCTTTTT

Sample #	Best identity	Species name	Best match	Sequence length	Sequence
26	1	Microcalanus pusillus	CAISN 950-13	313	TCTTTCAAGAAATATTGCACACGCAGGGGGCTCAGTAGA<br>TTTTGCTATTTTTTCTCTACATTTGGCCGGGGTGAGATCA<br>ATTTTAGGGGCAGTTAATTTTATTAGGACTTTAGGAAACC<br>TGCGAGTCTTTGGTATATTACTGGACCGGATACCATTATT<br>CGCATGGTCTGTCTTAGTCACTGCGGTGCTGCTATTGCTC<br>TCTATGCCAGTACTCGCTGGCGCGATTACTATGCTTCTAA<br>CAGACCGAAATTTAAACACGTCGTTTTATGATGTGGGAG<br>GGGGTGGAGACCCTATCTTATACCAACATCTTTTT
27	1	Microcalanus pusillus	CAISN 950-13	313	TCTTTCAAGAAATATTGCACACGCAGGGGGCTCAGTAGA<br>TTTTGCTATTTTTTCTCTACATTTGGCCGGGGTGAGATCA<br>ATTTTAGGGGCAGTTAATTTTATTAGGACTTTAGGAAACC<br>TGCGAGTCTTTGGTATATTACTGGACCGGATACCATTATT<br>CGCATGGTCTGTCTTAGTCACTGCGGTGCTGCTATTGCTC<br>TCTATGCCAGTACTCGCTGGCGCGATTACTATGCTTCTAA<br>CAGACCGAAATTTAAACACGTCGTTTTATGATGTGGGAG<br>GGGGTGGAGACCCTATCTTATACCAACATCTTTTT
28	1	Microcalanus pusillus	CAISN 950-13	313	TCTTTCAAGAAATATTGCACACGCAGGGGGCTCAGTAGA<br>TTTTGCTATTTTTTCTCTACATTTGGCCGGGGTGAGATCA<br>ATTTTAGGGGCAGTTAATTTTATTAGGACTTTAGGAAACC<br>TGCGAGTCTTTGGTATATTACTGGACCGGATACCATTATT<br>CGCATGGTCTGTCTTAGTCACTGCGGTGCTGCTATTGCTC<br>TCTATGCCAGTACTCGCTGGCGCGATTACTATGCTTCTAA<br>CAGACCGAAATTTAAACACGTCGTTTTATGATGTGGGAG<br>GGGGTGGAGACCCTATCTTATACCAACATCTTTTT

Sample #	Best identity	Species name	Best match	Sequence length	Sequence
29	1	Microcalanus pusillus	CAISN 950-13	313	TCTTTCAAGAAATATTGCACACGCAGGGGGCTCAGTAGATTTTGCTATTTTTTCTCTACATTTGGCCGGGGTGAGATCAATTTTAGGGGCAGTTAATTTTATTAGGACTTTAGGAAACCTGCGAGTCTTTGGTATATTACTGGACCGGATACCATTATTCGCATGGTCTGTCTTAGTCACTGCGGTGCTGCTATTGCTCTCTATGCCAGTACTCGCTGGCGCGATTACTATGCTTCTAACAGACCGAAATTTAAACACGTCGTTTTATGATGTGGGAGGGGGTGGAGACCCTATCTTATACCAACATCTTTTT
30	1	Microcalanus pusillus	CAISN 950-13	313	TCTTTCAAGAAATATTGCACACGCAGGGGGCTCAGTAGATTTTGCTATTTTTTCTCTACATTTGGCCGGGGTGAGATCAATTTTAGGGGCAGTTAATTTTATTAGGACTTTAGGAAACCTGCGAGTCTTTGGTATATTACTGGACCGGATACCATTATTCGCATGGTCTGTCTTAGTCACTGCGGTGCTGCTATTGCTCTCTATGCCAGTACTCGCTGGCGCGATTACTATGCTTCTAACAGACCGAAATTTAAACACGTCGTTTTATGATGTGGGAGGGGGTGGAGACCCTATCTTATACCAACATCTTTTT
31	1	Microcalanus pusillus	CAISN 950-13	313	TCTTTCAAGAAATATTGCACACGCAGGGGGCTCAGTAGATTTTGCTATTTTTTCTCTACATTTGGCCGGGGTGAGATCAATTTTAGGGGCAGTTAATTTTATTAGGACTTTAGGAAACCTGCGAGTCTTTGGTATATTACTGGACCGGATACCATTATTCGCATGGTCTGTCTTAGTCACTGCGGTGCTGCTATTGCTCTCTATGCCAGTACTCGCTGGCGCGATTACTATGCTTCTAACAGACCGAAATTTAAACACGTCGTTTTATGATGTGGGAGGGGGTGGAGACCCTATCTTATACCAACATCTTTTT

Sample #	Best identity	Species name	Best match	Sequence length	Sequence
32	0.996805112	Microcalanus pusillus	CAISN 950-13	313	TCTTTCAAGAAATACTGCACACGCAGGGGGCTCAGTAGA<br>TTTTGCTATTTTTTCTCTACATTTGGCCGGGGTGAGATCA<br>ATTTTAGGGGCAGTTAATTTTATTAGGACTTTAGGAAACC<br>TGCGAGTCTTTGGTATATTACTGGACCGGATACCATTATT<br>CGCATGGTCTGTCTTAGTCACTGCGGTGCTGCTATTGCTC<br>TCTATGCCAGTACTCGCTGGCGCGATTACTATGCTTCTAA<br>CAGACCGAAATTTAAACACGTCGTTTTATGATGTGGGAG<br>GGGGTGGAGACCCTATCTTATACCAACATCTTTTT
33	1	Microcalanus pusillus	CAISN 950-13	313	TCTTTCAAGAAATATTGCACACGCAGGGGGCTCAGTAGA<br>TTTTGCTATTTTTTCTCTACATTTGGCCGGGGTGAGATCA<br>ATTTTAGGGGCAGTTAATTTTATTAGGACTTTAGGAAACC<br>TGCGAGTCTTTGGTATATTACTGGACCGGATACCATTATT<br>CGCATGGTCTGTCTTAGTCACTGCGGTGCTGCTATTGCTC<br>TCTATGCCAGTACTCGCTGGCGCGATTACTATGCTTCTAA<br>CAGACCGAAATTTAAACACGTCGTTTTATGATGTGGGAG<br>GGGGTGGAGACCCTATCTTATACCAACATCTTTTT
34	1	Microcalanus pusillus	CAISN 950-13	313	TCTTTCAAGAAATATTGCACACGCAGGGGGCTCAGTAGA<br>TTTTGCTATTTTTTCTCTACATTTGGCCGGGGTGAGATCA<br>ATTTTAGGGGCAGTTAATTTTATTAGGACTTTAGGAAACC<br>TGCGAGTCTTTGGTATATTACTGGACCGGATACCATTATT<br>CGCATGGTCTGTCTTAGTCACTGCGGTGCTGCTATTGCTC<br>TCTATGCCAGTACTCGCTGGCGCGATTACTATGCTTCTAA<br>CAGACCGAAATTTAAACACGTCGTTTTATGATGTGGGAG<br>GGGGTGGAGACCCTATCTTATACCAACATCTTTTT

Sample #	Best identity	Species name	Best match	Sequence length	Sequence
35	0.996805112	Microcalanus pusillus	CAISN 950-13	313	TCTTTCAAGAAATATTGCACACGCAGGGGGCTCAGTAGA<br>TTTTGCTATTTTTTCTCTACATTTGGCCGGGGTGAGATCA<br>ATTTTAGGGGCAGTTAATTTTATTAGGACTTTAGGAAACC<br>TGCGAGTCTTTGGTATATTACTGGACCGGATACCATTATT<br>CGCATGGTCTGTCTTAGTCACTGCGGTGCTGCTATTGCTC<br>TCTATGCCAGTACTCGCTGGTGCGATTACTATGCTTCTAA<br>CAGACCGAAATTTAAACACGTCGTTTTATGATGTGGGAG<br>GGGGTGGAGACCCTATCTTATACCAACATCTTTTT
36	1	Microcalanus pusillus	CAISN 950-13	313	TCTTTCAAGAAATATTGCACACGCAGGGGGCTCAGTAGA<br>TTTTGCTATTTTTTCTCTACATTTGGCCGGGGTGAGATCA<br>ATTTTAGGGGCAGTTAATTTTATTAGGACTTTAGGAAACC<br>TGCGAGTCTTTGGTATATTACTGGACCGGATACCATTATT<br>CGCATGGTCTGTCTTAGTCACTGCGGTGCTGCTATTGCTC<br>TCTATGCCAGTACTCGCTGGCGCGATTACTATGCTTCTAA<br>CAGACCGAAATTTAAACACGTCGTTTTATGATGTGGGAG<br>GGGGTGGAGACCCTATCTTATACCAACATCTTTTT
37	1	Microcalanus pusillus	CAISN 950-13	313	TCTTTCAAGAAATATTGCACACGCAGGGGGCTCAGTAGA<br>TTTTGCTATTTTTTCTCTACATTTGGCCGGGGTGAGATCA<br>ATTTTAGGGGCAGTTAATTTTATTAGGACTTTAGGAAACC<br>TGCGAGTCTTTGGTATATTACTGGACCGGATACCATTATT<br>CGCATGGTCTGTCTTAGTCACTGCGGTGCTGCTATTGCTC<br>TCTATGCCAGTACTCGCTGGCGCGATTACTATGCTTCTAA<br>CAGACCGAAATTTAAACACGTCGTTTTATGATGTGGGAG<br>GGGGTGGAGACCCTATCTTATACCAACATCTTTTT

Sample #	Best identity	Species name	Best match	Sequence length	Sequence
38	1	Microcalanus pusillus	CAISN 950-13	313	TCTTTCAAGAAATATTGCACACGCAGGGGGCTCAGTAGA TTTTGCTATTTTTTCTCTACATTTGGCCGGGGTGAGATCA ATTTTAGGGGCAGTTAATTTTATTAGGACTTTAGGAAACC TGCGAGTCTTTGGTATATTACTGGACCGGATACCATTATT CGCATGGTCTGTCTTAGTCACTGCGGTGCTGCTATTGCTC TCTATGCCAGTACTCGCTGGCGCGATTACTATGCTTCTAA CAGACCGAAATTTAAACACGTCGTTTTATGATGTGGGAG GGGGTGGAGACCCTATCTTATACCAACATCTTTTT
39	0.996805112	Microcalanus pusillus	CAISN 950-13	313	TCTTTCAAGAAATATTGCACACGCAGGGGGCTCAGTAGA TTTTGCTATTTTTTCTCTACATTTGGCCGGGGTGAGATCA ATTTTAGGGGCAGTTAATTTTATTAGGACTTTAGGAAACC TGCGAGTCTTTGGTATATTACTGGACCGGATACCATTATT CGCATGGTCTGTCTTAGTCACTGCGGTGCTGCTATTGCTC TCTATGCCAGTACTCGCTGGTGCGATTACTATGCTTCTAA CAGACCGAAATTTAAACACGTCGTTTTATGATGTGGGAG GGGGTGGAGACCCTATCTTATACCAACATCTTTTT
40	1	Microcalanus pusillus	CAISN 950-13	313	TCTTTCAAGAAATATTGCACACGCAGGGGGCTCAGTAGA TTTTGCTATTTTTTCTCTACATTTGGCCGGGGTGAGATCA ATTTTAGGGGCAGTTAATTTTATTAGGACTTTAGGAAACC TGCGAGTCTTTGGTATATTACTGGACCGGATACCATTATT CGCATGGTCTGTCTTAGTCACTGCGGTGCTGCTATTGCTC TCTATGCCAGTACTCGCTGGCGCGATTACTATGCTTCTAA CAGACCGAAATTTAAACACGTCGTTTTATGATGTGGGAG GGGGTGGAGACCCTATCTTATACCAACATCTTTTT

Sample #	Best identity	Species name	Best match	Sequence length	Sequence
41	1	Microcalanus pusillus	CAISN 950-13	313	TCTTTCAAGAAATATTGCACACGCAGGGGGCTCAGTAGA<br>TTTTGCTATTTTTTCTCTACATTTGGCCGGGGTGAGATCA<br>ATTTTAGGGGCAGTTAATTTTATTAGGACTTTAGGAAACC<br>TGCGAGTCTTTGGTATATTACTGGACCGGATACCATTATT<br>CGCATGGTCTGTCTTAGTCACTGCGGTGCTGCTATTGCTC<br>TCTATGCCAGTACTCGCTGGCGCGATTACTATGCTTCTAA<br>CAGACCGAAATTTAAACACGTCGTTTTATGATGTGGGAG<br>GGGGTGGAGACCCTATCTTATACCAACATCTTTTT
42	1	Microcalanus pusillus	CAISN 950-13	313	TCTTTCAAGAAATATTGCACACGCAGGGGGCTCAGTAGA<br>TTTTGCTATTTTTTCTCTACATTTGGCCGGGGTGAGATCA<br>ATTTTAGGGGCAGTTAATTTTATTAGGACTTTAGGAAACC<br>TGCGAGTCTTTGGTATATTACTGGACCGGATACCATTATT<br>CGCATGGTCTGTCTTAGTCACTGCGGTGCTGCTATTGCTC<br>TCTATGCCAGTACTCGCTGGCGCGATTACTATGCTTCTAA<br>CAGACCGAAATTTAAACACGTCGTTTTATGATGTGGGAG<br>GGGGTGGAGACCCTATCTTATACCAACATCTTTTT
43	1	Microcalanus pusillus	CAISN 950-13	313	TCTTTCAAGAAATATTGCACACGCAGGGGGCTCAGTAGA<br>TTTTGCTATTTTTTCTCTACATTTGGCCGGGGTGAGATCA<br>ATTTTAGGGGCAGTTAATTTTATTAGGACTTTAGGAAACC<br>TGCGAGTCTTTGGTATATTACTGGACCGGATACCATTATT<br>CGCATGGTCTGTCTTAGTCACTGCGGTGCTGCTATTGCTC<br>TCTATGCCAGTACTCGCTGGCGCGATTACTATGCTTCTAA<br>CAGACCGAAATTTAAACACGTCGTTTTATGATGTGGGAG<br>GGGGTGGAGACCCTATCTTATACCAACATCTTTTT

Sample #	Best identity	Species name	Best match	Sequence length	Sequence
44	1	Microcalanus pusillus	CAISN 950-13	313	TCTTTCAAGAAATATTGCACACGCAGGGGGCTCAGTAGA TTTTGCTATTTTTTTCTCTACATTTGGCCGGGGTGAGATCA ATTTTAGGGGCAGTTAATTTTATTAGGACTTTAGGAAACC TGCGAGTCTTTGGTATATTACTGGACCGGATACCATTATT CGCATGGTCTGTCTTAGTCACTGCGGTGCTGCTATTGCTC TCTATGCCAGTACTCGCTGGCGCGATTACTATGCTTCTAA CAGACCGAAATTTAAACACGTCGTTTTATGATGTGGGAG GGGGTGGAGACCCTATCTTATACCAACATCTTTTT
45	0.996805112	Microcalanus pusillus	CAISN 950-13	313	TCTTTCAAGAAATATTGCACACGCAGGGGGCTCAGTAGA TTTTGCTATTTTTTTCTCTACATTTGGCCGGGGTAAGATCA ATTTTAGGGGCAGTTAATTTTATTAGGACTTTAGGAAACC TGCGAGTCTTTGGTATATTACTGGACCGGATACCATTATT CGCATGGTCTGTCTTAGTCACTGCGGTGCTGCTATTGCTC TCTATGCCAGTACTCGCTGGCGCGATTACTATGCTTCTAA CAGACCGAAATTTAAACACGTCGTTTTATGATGTGGGAG GGGGTGGAGACCCTATCTTATACCAACATCTTTTT
46	1	Microcalanus pusillus	CAISN 950-13	313	TCTTTCAAGAAATATTGCACACGCAGGGGGCTCAGTAGA TTTTGCTATTTTTTTCTCTACATTTGGCCGGGGTGAGATCA ATTTTAGGGGCAGTTAATTTTATTAGGACTTTAGGAAACC TGCGAGTCTTTGGTATATTACTGGACCGGATACCATTATT CGCATGGTCTGTCTTAGTCACTGCGGTGCTGCTATTGCTC TCTATGCCAGTACTCGCTGGCGCGATTACTATGCTTCTAA CAGACCGAAATTTAAACACGTCGTTTTATGATGTGGGAG GGGGTGGAGACCCTATCTTATACCAACATCTTTTT

Sample #	Best identity	Species name	Best match	Sequence length	Sequence
47	1	Microcalanus pusillus	CAISN 950-13	313	TCTTTCAAGAAATATTGCACACGCAGGGGGCTCAGTAGA TTTTGCTATTTTTTCTCTACATTTGGCCGGGGTGAGATCA ATTTTAGGGGCAGTTAATTTTATTAGGACTTTAGGAAACC TGCGAGTCTTTGGTATATTACTGGACCGGATACCATTATT CGCATGGTCTGTCTTAGTCACTGCGGTGCTGCTATTGCTC TCTATGCCAGTACTCGCTGGCGCGATTACTATGCTTCTAA CAGACCGAAATTTAAACACGTCGTTTTATGATGTGGGAG GGGGTGGAGACCCTATCTTATACCAACATCTTTTT
48	1	Microcalanus pusillus	CAISN 950-13	313	TCTTTCAAGAAATATTGCACACGCAGGGGGCTCAGTAGA TTTTGCTATTTTTTCTCTACATTTGGCCGGGGTGAGATCA ATTTTAGGGGCAGTTAATTTTATTAGGACTTTAGGAAACC TGCGAGTCTTTGGTATATTACTGGACCGGATACCATTATT CGCATGGTCTGTCTTAGTCACTGCGGTGCTGCTATTGCTC TCTATGCCAGTACTCGCTGGCGCGATTACTATGCTTCTAA CAGACCGAAATTTAAACACGTCGTTTTATGATGTGGGAG GGGGTGGAGACCCTATCTTATACCAACATCTTTTT
49	1	Microcalanus pusillus	CAISN 950-13	313	TCTTTCAAGAAATATTGCACACGCAGGGGGCTCAGTAGA TTTTGCTATTTTTTCTCTACATTTGGCCGGGGTGAGATCA ATTTTAGGGGCAGTTAATTTTATTAGGACTTTAGGAAACC TGCGAGTCTTTGGTATATTACTGGACCGGATACCATTATT CGCATGGTCTGTCTTAGTCACTGCGGTGCTGCTATTGCTC TCTATGCCAGTACTCGCTGGCGCGATTACTATGCTTCTAA CAGACCGAAATTTAAACACGTCGTTTTATGATGTGGGAG GGGGTGGAGACCCTATCTTATACCAACATCTTTTT

Sample #	Best identity	Species name	Best match	Sequence length	Sequence
50	1	Microcalanus pusillus	CAISN 950-13	313	TCTTTCAAGAAATATTGCACACGCAGGGGGCTCAGTAGATTTTGCTATTTTTTCTCTACATTTGGCCGGGGTGAGATCAATTTTAGGGGCAGTTAATTTTATTAGGACTTTAGGAAACCTGCGAGTCTTTGGTATATTACTGGACCGGATACCATTATTCGCATGGTCTGTCTTAGTCACTGCGGTGCTGCTATTGCTCTCTATGCCAGTACTCGCTGGCGCGATTACTATGCTTCTAACAGACCGAAATTTAAACACGTCGTTTTATGATGTGGGAGGGGGTGGAGACCCTATCTTATACCAACATCTTTTT
51	1	Microcalanus pusillus	CAISN 950-13	313	TCTTTCAAGAAATATTGCACACGCAGGGGGCTCAGTAGATTTTGCTATTTTTTCTCTACATTTGGCCGGGGTGAGATCAATTTTAGGGGCAGTTAATTTTATTAGGACTTTAGGAAACCTGCGAGTCTTTGGTATATTACTGGACCGGATACCATTATTCGCATGGTCTGTCTTAGTCACTGCGGTGCTGCTATTGCTCTCTATGCCAGTACTCGCTGGCGCGATTACTATGCTTCTAACAGACCGAAATTTAAACACGTCGTTTTATGATGTGGGAGGGGGTGGAGACCCTATCTTATACCAACATCTTTTT
52	1	Microcalanus pusillus	CAISN 950-13	313	TCTTTCAAGAAATATTGCACACGCAGGGGGCTCAGTAGATTTTGCTATTTTTTCTCTACATTTGGCCGGGGTGAGATCAATTTTAGGGGCAGTTAATTTTATTAGGACTTTAGGAAACCTGCGAGTCTTTGGTATATTACTGGACCGGATACCATTATTCGCATGGTCTGTCTTAGTCACTGCGGTGCTGCTATTGCTCTCTATGCCAGTACTCGCTGGCGCGATTACTATGCTTCTAACAGACCGAAATTTAAACACGTCGTTTTATGATGTGGGAGGGGGTGGAGACCCTATCTTATACCAACATCTTTTT

Sample #	Best identity	Species name	Best match	Sequence length	Sequence
53	0.996805112	Microcalanus pusillus	CAISN 950-13	313	TCTTTCAAGAAATATTGCACACGCAGGGGGCTCAGTAGATTTTGCTATTTTTTCTCTACATTTGGCCGGGGTGAGATCAATTTTAGGGGCAGTTAATTTTATTAGGACTTTAGGAAACCTGCGAGTATTTGGTATATTACTGGACCGGATACCATTATTCGCATGGTCTGTCTTAGTCACTGCGGTGCTGCTATTGCTCTCTATGCCAGTACTCGCTGGCGCGATTACTATGCTTCTAACAGACCGAAATTTAAACACGTCGTTTTATGATGTGGGAGGGGGTGGAGACCCTATCTTATACCAACATCTTTTT
54	1	Microcalanus pusillus	CAISN 950-13	313	TCTTTCAAGAAATATTGCACACGCAGGGGGCTCAGTAGATTTTGCTATTTTTTCTCTACATTTGGCCGGGGTGAGATCAATTTTAGGGGCAGTTAATTTTATTAGGACTTTAGGAAACCTGCGAGTCTTTGGTATATTACTGGACCGGATACCATTATTCGCATGGTCTGTCTTAGTCACTGCGGTGCTGCTATTGCTCTCTATGCCAGTACTCGCTGGCGCGATTACTATGCTTCTAACAGACCGAAATTTAAACACGTCGTTTTATGATGTGGGAGGGGGTGGAGACCCTATCTTATACCAACATCTTTTT
55	1	Microcalanus pusillus	CAISN 950-13	313	TCTTTCAAGAAATATTGCACACGCAGGGGGCTCAGTAGATTTTGCTATTTTTTCTCTACATTTGGCCGGGGTGAGATCAATTTTAGGGGCAGTTAATTTTATTAGGACTTTAGGAAACCTGCGAGTCTTTGGTATATTACTGGACCGGATACCATTATTCGCATGGTCTGTCTTAGTCACTGCGGTGCTGCTATTGCTCTCTATGCCAGTACTCGCTGGCGCGATTACTATGCTTCTAACAGACCGAAATTTAAACACGTCGTTTTATGATGTGGGAGGGGGTGGAGACCCTATCTTATACCAACATCTTTTT

Sample #	Best identity	Species name	Best match	Sequence length	Sequence
56	1	Microcalanus pusillus	CAISN 950-13	313	TCTTTCAAGAAATATTGCACACGCAGGGGGCTCAGTAGATTTTGCTATTTTTTCTCTACATTTGGCCGGGGTGAGATCAATTTTAGGGGCAGTTAATTTTATTAGGACTTTAGGAAACCTGCGAGTCTTTGGTATATTACTGGACCGGATACCATTATTCGCATGGTCTGTCTTAGTCACTGCGGTGCTGCTATTGCTCTCTATGCCAGTACTCGCTGGCGCGATTACTATGCTTCTAACAGACCGAAATTTAAACACGTCGTTTTATGATGTGGGAGGGGGTGGAGACCCTATCTTATACCAACATCTTTTT
57	1	Microcalanus pusillus	CAISN 950-13	313	TCTTTCAAGAAATATTGCACACGCAGGGGGCTCAGTAGATTTTGCTATTTTTTCTCTACATTTGGCCGGGGTGAGATCAATTTTAGGGGCAGTTAATTTTATTAGGACTTTAGGAAACCTGCGAGTCTTTGGTATATTACTGGACCGGATACCATTATTCGCATGGTCTGTCTTAGTCACTGCGGTGCTGCTATTGCTCTCTATGCCAGTACTCGCTGGCGCGATTACTATGCTTCTAACAGACCGAAATTTAAACACGTCGTTTTATGATGTGGGAGGGGGTGGAGACCCTATCTTATACCAACATCTTTTT
58	0.996805112	Microcalanus pusillus	CAISN 950-13	313	TCTTTCAAGAAATATTGCACACGCAGGGGGCTCAGTAGATTTTGCTATTTTTTCTCTACATTTGGCCGGGGTGAGATCAATTTTAGGGGCAGTTAATTTTATTAGGACTTTAGGAAACCTGCGAGTCTTTGGTATATTACTGGACCGGATACCATTATTCGCATGGTCTGTCTTAGTCACTGCGGTGCTGCTATTGCTCTCTATGCCAGTACTCGCTGGCGCGATTACTATGCTTCTAACAGACCGAAATTTAAACACGTCGTTTTATGATGTGGGAGGGGGTGGAGATCCTATCTTATACCAACATCTTTTT
